# A Graphene/MXene-Modified Flexible Fabric for Infrared Camouflage, Electrothermal, and Electromagnetic Interference Shielding

**DOI:** 10.3390/nano15020098

**Published:** 2025-01-09

**Authors:** Xianguang Hou, Ziyi Zang, Yaxin Meng, Tian Wang, Shuai Gao, Qingman Liu, Lijun Qu, Xiansheng Zhang

**Affiliations:** 1 Shandong Key Laboratory of Medical and Health Textile Materials, Qingdao University, Qingdao 266071, China; houxianguang@qdu.edu.cn (X.H.); zangziyi@qdu.edu.cn (Z.Z.); 2021020761@qdu.edu.cn (Y.M.); wangtian@qdu.edu.cn (T.W.); gaoshuai@qdu.edu.cn (S.G.); liuqingman@qdu.edu.cn (Q.L.); lijunqu@qdu.edu.cn (L.Q.); 2 College of Textiles and Clothing, Qingdao University, Qingdao 266071, China; 3 Sichuan Provincial Engineering Research Center of Functional Development and Application of High Performance Special Textile Materials (Chengdu Textile College), Chengdu 611731, China

**Keywords:** MXene, graphene, infrared camouflage, electrothermal, EMI shield

## Abstract

Although materials with infrared camouflage capabilities are increasingly being produced, few applications exist in clothing fabrics. Here, graphene/MXene-modified fabric with superior infrared camouflage, Joule heating, and electromagnetic shielding capabilities all in one was prepared by simply scraping a graphene slurry onto alkali-treated cotton fabrics, followed by spraying MXene. The functionality of the modified fabrics after different treatment times was then tested and analyzed. The results indicate that the mid-infrared emissivity of the modified fabric decreases with an increase in the coating times of graphene and MXene. When the graphene/MXene-modified fabrics are prepared at loads of 5 and 1.2 mg/cm^2^, respectively, the modified fabrics have very low infrared emissivity in the 3–5 and 8–14 μm bands, and the surface temperature can be reduced by 53.1 °C when placed on a heater with a temperature of 100 °C (surface radiation temperature of 95 °C). The modified fabric also demonstrates excellent Joule heating capabilities; at 4 V of power, a temperature of 91.7 °C may be reached in 30 s. In addition, customized materials exhibit strong electromagnetic shielding performance. By simply folding the cloth, the electromagnetic interference shield effect can be increased to 64.3 dB. With their superior infrared camouflage, thermal management, and electromagnetic shielding performance, graphene/MXene-modified fabrics have found extensive use in intelligent wearables and military applications.

## 1. Introduction

Superior hiding ability is frequently a crucial factor in winning a war. Hence, anti-detection technology should be enhanced alongside ongoing advances in detection technology. Among these is infrared (IR) stealth technology, which shields or reduces an object’s IR radiation, making it nearly identical to the background radiation of the surrounding area, thus rendering it undetectable to IR sensors and achieving a stealth effect [1]. However, several problems exist with IR stealth technology. As IR detection technology advances, IR stealth technology must continually enhance its performance to meet increasingly demanding detection environments. This technology is intended to stop hostile IR sensors from detecting targets and is frequently utilized in the military [2]. Nonetheless, it is commonly recognized that any item warmer than absolute zero radiates IR light in all directions [3]. IR detection has two atmospheric windows [4] (3–5 μm for medium-wave IR and 8–14 μm for long-wave infrared) due to variations in electromagnetic wave propagation attenuation. Consequently, for military applications, it is crucial to lower the exterior IR emission of items in these two bands. Stefan–Boltzmann’s theorem, *E = εσT*^4^, where *E* is the radiant exitance or emittance of a black body, measured in watts per square meter (W/m²); *ε* is the emissivity of the surface, a dimensionless number between 0 and 1, which indicates how closely the object’s emission behavior approaches that of an ideal black body; *σ* is the Stefan–Boltzmann constant, approximately equal to 5.67 × 10^−8^ W/(m^2^·K^4^); and *T* is the absolute temperature of the body in kelvins (K). The theorem states that an object’s absolute temperature and IR emissivity are the primary determinants of IR stealth [5]. Thus, for a given coefficient σ, lowering the absolute temperature T and IR emissivity ε will effectively reduce IR emission [6,7,8]. Therefore, combining materials with low IR emissivity and thermal insulation capability effectively reduces IR radiation.

Few natural low-emissivity materials have been explored, and the more established magnetron-sputtered metallic materials on object surfaces have excellent low-IR emissivity properties but are significantly affected by surface smoothness [9]. Other methods to reduce IR emissivity include the use of phase change materials [10], metamaterials [11,12], photonic crystals [13], coated materials [14], or composites with fillers such as conjugated polymers [15], carbon [16], and polar oxides [17]. Unfortunately, these materials’ high preparation costs and technical constraints significantly restrict their utilization.

Personal thermal management (PTM) can be divided into two categories: active and passive. In order to heat or cool garments, active technologies primarily rely on external energy sources [18,19], such as electrical energy. For instance, electrical energy is used to maintain body temperature in frigid climates, allowing for all-weather thermal regulation of the human body [20]. Such technologies typically come with higher energy consumption and prices, as well as potential mechanical complexity and weight difficulties, even though they enable higher thermal management efficiencies and a wider range of application settings. However, in warfare, one needs the most advanced camouflage technology and strategies to disorient the adversary. The superior Joule thermal properties can provide a practical means to generate the thermal infrared detection of false targets during armed conflict. Passive technologies, on the other hand, mainly rely on the physical properties of materials to realize thermal management, such as thermal insulating materials and reflective materials, without relying on an external power source. An example is the use of aerogel-based materials [21,22] as thermal insulators, which are widely used in personal thermal management due to their low thermal conductivity, thermal stability, and high insulating properties. Passive technologies are usually more environmentally friendly and economical, but they may not be able to achieve the same fast and precise temperature regulation as active technologies.

The continuous development of electronic products with high integration and high power density has aroused widespread concern about electromagnetic interference (EMI) [23,24]. Electromagnetic radiation generated by electronic equipment will not only lead to the impact of the surrounding electronic equipment but also directly threaten human health, so it is particularly important to develop protective equipment with high EMI shielding performance [25,26,27].

Two-dimensional (2D) material MXene (Ti_3_C_2_T_x_) is a class of 2D materials that were added to the 2D family in 2011 [28]. It is commonly expressed as M_n_ + X_n_T_x_, where M stands for transition metal atoms, X for C or/and N, T_x_ for surface termination, and n for the number of atomic layers. MXene typically comprises multiple layers of transition metal carbides or nitrides, separated alternately by intermetallic layers and layers of carbon or nitrogen. The (-OH, -O, and -F) functional groups on the surface of MXene nanosheets give them metallic conductivity and good mechanical properties, so they are widely used to construct high-performance infrared stealth and electromagnetic interference shielding materials. The internal electrons of MXene transition under the action of infrared photon energy, converting the infrared energy into the energy of electrons, so that the infrared radiation is absorbed and the infrared energy radiated outward is reduced [29,30,31]. MXene has excellent gold properties and high conductivity. When the electromagnetic wave is incident, the free electron oscillates under the action of alternating electromagnetic field, forming a current, generating a reflected wave in the opposite direction of the incident wave so that part of the electromagnetic wave is reflected back and cannot enter the inside of the material to achieve electromagnetic shielding [32]. Li et al. [33] treated Ti_3_C_2_T_x_ MXene film with 0.04 M hydrochloric acid, resulting in an electrical conductivity of 3584 Scm^−1^ and EMISE of 66.2 dB and significantly improved mechanical properties. Hassan et al. [34] used vacuum-assisted filtration to prepare conductive, flexible, robust, and versatile MXene/CNT Janus films. It exhibits an efficient and stable EMI shielding effect of 72 dB in the X-band, with excellent thermal camouflage performance over a wide temperature range of −1 to 300 °C.

This work reports a practical modified fabric with outstanding electrothermal properties (4 V, 91.7 °C), good electromagnetic shielding properties (64.3 dB), and low IR emissivity (0.248). These properties may be industrialized by basic screen printing and spraying techniques. This study utilized graphene paste, comprising graphene and carbon nanotubes, to act as a transverse thermal conductive layer, uniformly dispersing the heat in the graphene layer and reducing the surface resistance of the fabric. To lower the cloth surface’s IR emissivity, MXene was applied as an IR low-emission layer [35]. Graphene and MXene coatings were applied to cotton fabric to create graphene/MXene-modified IR stealth fabric. Modified fabrics with excellent heat radiation blocking performance can not only be used to prevent infrared detection but can also be used to make warm clothing so that people in a cold environment can block human body heat emission and achieve passive warmth. They can also be electrically heated to play an active role in warmth. The efficient electromagnetic shielding performance of modified fabrics can help people or objects shield the electromagnetic interference of the surrounding environment, such as pregnant women, workers in complex electromagnetic interference environments, and high-precision instruments. This project aims to develop a novel technique and concept for large-scale creating long-lasting, useful graphene/MXene-modified fabrics with superior IR stealth, electrothermal properties, and electromagnetic shielding qualities. These materials are anticipated to be crucial in developing smart wearables and military applications.

## 2. Materials and Methods

### 2.1. Materials

Jinan Sanchuan New Material Technology Co., Ltd. (Jinan, China) provided Ti_3_C_2_T_x_ MXene aqueous suspension (20 mg/mL). Jiangnan Graphene Research Institute provided physical stripping graphene. Suqian Nakate New Material Technology Co., Ltd. (Suqian, China) provided carbon nanotube aqueous suspension (10 wt%) and silane-modified acrylic resin ((C_3_H_6_O)_n_C_14_H_32_N_2_O_9_Si_2_) (25 wt%). Thickeners (acrylic aqueous emulsion, 30 wt%) and Cotton twill fabric (0.6 mm thick) were provided by Qingdao XueDa Group Co. (Qingdao, China).

### 2.2. Preparation of Sample Cloth

The sample cloth was placed in a beaker containing a solution of Na_2_CO_3_ (6 g/L) and NaOH (15 g/L) and heated in a water bath at 75 °C for 30 min. It was then rinsed with water to neutralize and dried in an oven at 65 °C to obtain cotton fabrics designated as C-1. Subsequently, the cotton fabrics were treated in plasma surface technology for 5 min at 800 W.

### 2.3. Preparation of Graphene Scratch Coating Paste

Graphene, carbon nanotubes, water, and binder (silane-modified acrylic resin (C_3_H_6_O)_n_C_14_H_32_N_2_O_9_Si_2_) were added to a stirrer in the same mass ratio and stirred at 4000 r/min for 2 h, making graphene paste. The graphene paste was then mixed with thickener at a mass ratio of 100:0.5 and stirred at 600 r/min for 10 min to produce graphene scratch coating paste.

### 2.4. Preparation of Sample

**Figure 1:**
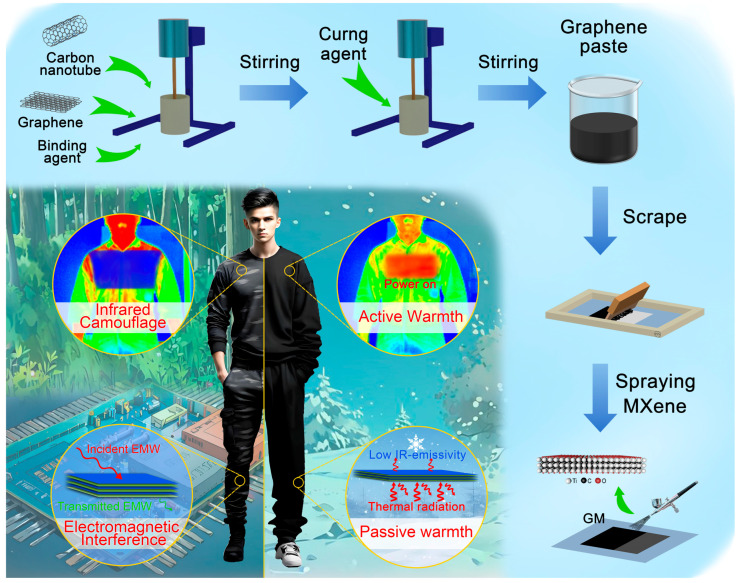
Preparation process and functional diagram of graphene/MXene-modified fabric.

The graphene squeegee paste was applied onto the cotton fabric (10 × 10 cm, twill side) C-1 by a screen-printing plate and dried in an oven at 40 °C for 60 min. Subsequently, 2 mL of MXene was sprayed on the graphene surface with an air compressor and placed in a drying oven at 40 °C for 30 min to obtain a graphene/MXene-modified fabric, noted as GM-1. The graphene/MXene-modified fabrics obtained by repeating this process one and two more times were named GM-2 and GM-3, respectively. The fabric obtained by scraping the graphene paste once on C-1 is denoted as G-1, and the fabric obtained by spraying only 2 mL of MXene on C-1 is denoted as M-1. The preparation process and functional diagram are shown schematically in Figure 1. The sample types and preparation method are shown in Table 1.

### 2.5. Research Methods

A field-emission scanning electron microscope instrument (SEM, Phenom Pro, Phenome, Eindhoven, The Netherlands) was used to describe the microstructures and morphologies of the obtained samples. A microcomputer-controlled electronic universal testing machine (WDW-2T, Jinan Heng Rui Jin Testing Machine Co., Ltd., Jinan, China) was used to measure the breaking tensile strength of fabrics at the speed of 25 mm and 50 mm/min across. X-ray diffractometer (XRD; Ultima IV, Rigaku, Akishima-shi, Tokyo, Japan) using Cu Kα radiation (λ = 1.5406 Å) was used to characterize the crystalline phase of C-1 and GM-1 fabrics at a scan rate of 5° min^−1^ from 5° to 90°. Elemental analysis of the samples was performed via an X-ray photoelectron spectrometer (XPS, Axis Supra+, Shimadzu, Kyoto, Japan). The IR photos were captured using an IR camera (FLIR A325sc, FLIR Systems, Wilsonville, Oregon, USA). A vector network analyzer (E5080B, Keysight Technologies, Malaysia) in the X band (8–12.4 GHz) was used to test the performance of the EMI shielding based on a waveguide method. A multifunction digital four-point tester was utilized to assess the modified fabric’s electrical conductivities at room temperature (ST-2258C, Suzhou Jingge Electronic Co., Ltd., Suzhou, China). Plasma surface technology was used to apply 800 W of power to treat the cotton fabric’s surface (LFG1000, Diener, Ebhausen, Germany). An adjustable direct current power supply provided stable and variable voltage to both ends of the modified fabric (MS-155D, MAISHENG, Dongguan, China). A microcomputer heating table was used to heat the sample (JF-956, JFTOOIS, Dongguan, China). Air compressors were used to spray the MXene solution onto cotton fabrics (UA-601G, U-STAR, Dongguan, China).

Tensile fracture strength test: The tensile fracture strength of a 1 × 5 cm sample is measured at a span of 25 mm and a speed of 50 mm per minute by clamping the short edge of the sample on an electronic universal testing machine controlled by a microcomputer.

Infrared camouflage test: a sample of size 3 × 3 cm is placed on the heating table (a ceramic plate is placed in the middle for uniform heating). The sample and the heating table are photographed with an infrared detector, and the difference in color displayed on the screen is the temperature difference between the two. The greater the difference in temperature, the better the thermal insulation effect.

Electrothermal performance test: a voltage was applied at both ends of the 1 × 2 cm-sized samples, and the voltage was controlled by adjusting the knob on the power supply box to 1.5, 2.0, 2.5, 3.0, 3.5, and 4.0 V. The radiant temperatures of the sample surfaces were observed by using a thermal imager.

Electromagnetic interference shielding performance test: the electromagnetic shielding test equipment was calibrated, then a 2.5 × 5 cm sample was placed in the center of the two ports, screwed down, and tested.

## 3. Results and Discussion

### 3.1. Characterization and Mechanical Properties of Graphene/MXene-Modified Fabrics

**Figure 2:**
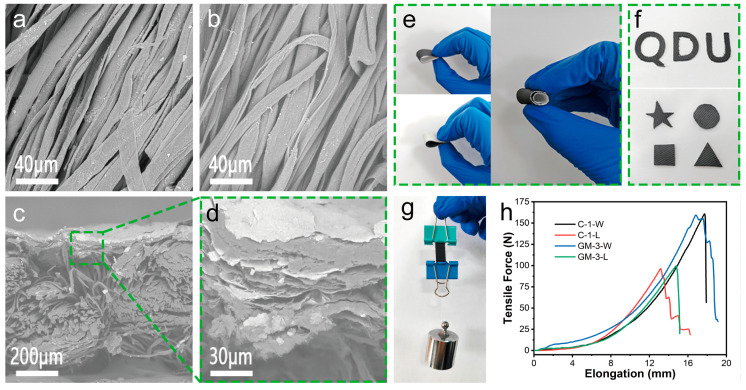
Structure and mechanical properties of graphene/MXene-modified fabrics. (**a**,**b**) C-1 SEM image of fabric surface before and after treatment; (**c**,**d**) SEM images of GM-3 fabric cross-section at different magnifications; (**e**) flexibility of modified fabrics; (**f**) cutting modified fabrics into different shapes; (**g**) heavy loading picture of GM-3 fabric; (**h**) tensile breaking strength testing of C-1 fabric and GM-3 fabric in the warp (W) and latitude (L) directions (the sample size is 1 × 5 cm).

Compared to Figure 2a, the results of alkali washing of cotton fabrics showed a significant reduction of impurities on the surface of cotton fabrics (Figure 2b). This helps in the attachment of graphene paste to the surface of cotton fabrics. The coating affixed to the C-1 cloth is only a thin layer, as seen in Figure 2c. A closer look at the enlarged image in Figure 2d reveals numerous delaminations within the coating and a sheet-like buildup of graphene and MXene within the layers. These gaps between the layers also somewhat obstruct heat conduction in the vertical direction and increase the reflection loss of electromagnetic waves transmitted to the coating’s interior.

The GM-3 fabric demonstrates good softness by its ability to be rolled and folded forward and backward (Figure 2e). Here, we mainly increase the fastness of the modified fabric by adding adhesives, and can also use special structures such as simulated fish scale design to effectively enhance the stress distribution and prevent excessive localized stress to increase the stability of the material [36]. Particularly noteworthy is its capacity to be cut into QDU, pentagram, circle, square, and triangle patterns (Figure 2f), demonstrating the fabric’s strong machinability and versatility in shape. Clamping both ends of the sample (1 × 4 cm), we attach a 100 g weight to one end of the clip and lift the other end by hand to directly reflect the strength of the sample (Figure 2g). The GM-3 textile exhibits remarkable mechanical strength, and the coating coverage does not compromise the cotton fabric’s inherent mechanical strength. As shown in Figure 2h, the tensile breaking strength of the original fabric was 160.669 N and 96.25 N in the warp and latitude directions, respectively, while the tensile breaking strength of the modified fabric was 159.209 N and 99.586 N in the warp and latitude directions, respectively. The test results showed that the modified fabric did not affect the mechanical properties of the original fabric (this can also be seen in Figure S1), which should be because, in the process of scraping or spraying the slurry, it was only loaded onto the surface of the original fabric in its physical form and did not damage the structure and chemical bonds of the original fabric. This should be due to the fact that during the scraping or spraying process, the paste was only physically loaded onto the surface of the original fabric and did not damage the structure or chemical bonding of the original fabric. The modified fabric has good fastness, and after soaking in water at 25 °C for 24 h, no objects were found to fall off the GM-3 fabric, as depicted in Figure S2.

**Figure 3:**
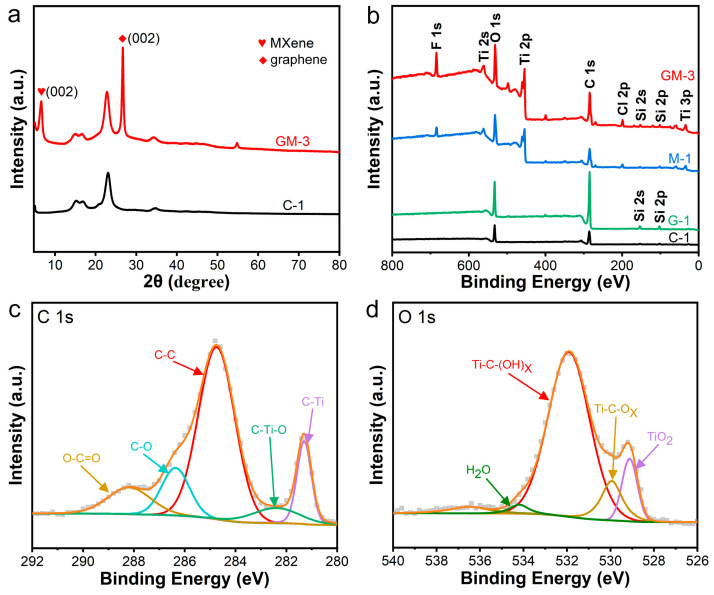
Characterization of graphene/MXene-modified fabrics. (**a**) XRD spectra of C-1 and GM-3 fabrics; (**b**) XPS spectra of C-1, G-1, M-1, and GM-3 fabrics; (**c**,**d**) C 1s and O 1s high-resolution spectra of GM-3 fabrics.

Figure 3a displays the XRD spectra of fabrics C-1 and GM-3. In addition to the original characteristic peak of the C-1 fabric, characteristic peaks of MXene (002) and graphene (002) were found at 2θ = 6.62° and 26.28°, respectively, indicating successful loading of graphene and MXene onto the C-1 fabric. The characteristic peak of graphite was found at 2θ = 54°, which may be due to some graphite not completely delaminated into a single layer during the physical exfoliation process. The wide-scan and high-resolution spectra of C 1s and O 1s obtained by XPS are displayed in Figure 3b–d. The C 1s peak, with a binding energy of 284.8 eV, serves as the reference for all spectra.

In addition, XPS was used to further analyze the surface elemental composition and chemical properties of GM-3 fabrics (Figure 3b). The results showed that Si 2p and Si 2s peaks appeared at binding energies of 102.7 eV and 154 eV compared to C-1 fabrics, indicating that the graphene paste contains silane-modified acrylic resin, which is crucial for the fastness of the graphene paste after scraping on the fabric. The presence of elements like C, O, Ti, F, and Cl on the GM-3 fabric. The presence of F and Cl elements possibly represents residual MAX phase corrosion with strong acid during the preparation of MXene. The detection of all MXene-related peaks in the M-1 and GM-3 fabrics suggests that the MXene structure was neither disrupted nor destroyed during the loading procedure. Additionally, C 1s can be decomposed to 288.2, 286.4, 284.7, 282.4, and 281.3 eV, corresponding to O–C=O, C–O, C–C, C–Ti–O, and C–Ti bond structures, respectively (Figure 3c). In particular, the presence of C–O bonds indicates oxygen-containing functional groups of amorphous carbon in GM-3 fabrics, which facilitates the formation of hydrogen bonds and the generation of dipole polarization, enhancing the material’s fastness [37,38]. The high-resolution O 1s spectra of the GM-3 fabric surface can be fitted with four peaks at 534.2, 531.9, 529.9, and 529.1 eV, corresponding to H_2_O, Ti–C–(OH)_X_, Ti–C–O_X_, and TiO_2_ bonding structures, respectively. Furthermore, the Ti 2p can be decomposed into 463.3, 460.6, 458.7, 457.4, and 454.4 eV, corresponding to Ti–O, C–Ti–F, TiO_2_, Ti–O, and Ti–C bond structures, respectively [39]. This indicates –OH and =O oxygen-containing functional groups on the fabric surface (Figure 3d and Figure S3).

### 3.2. Infrared Camouflage Properties of Graphene/MXene-Modified Fabrics

**Figure 4:**
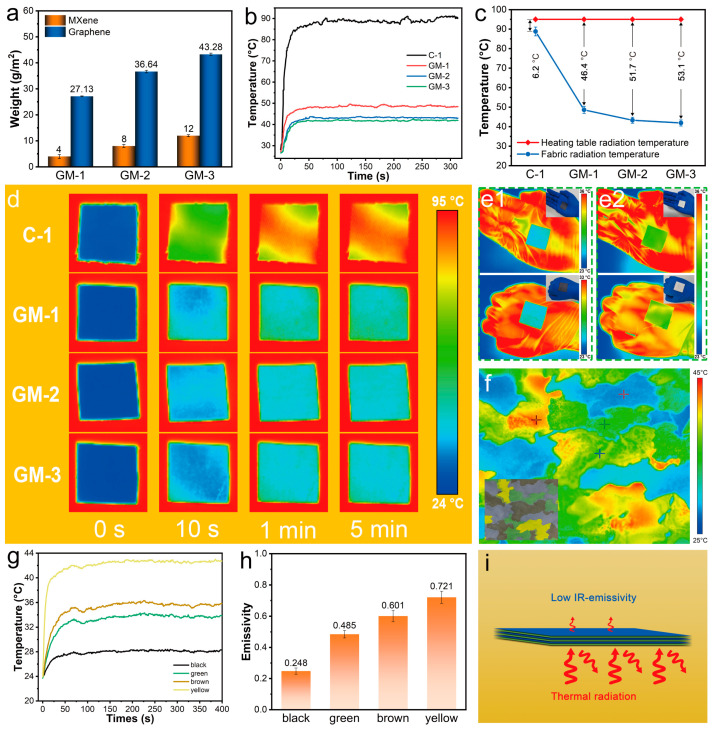
IR stealth performance of graphene/MXene-modified IR stealth fabric. (**a**) GM-1, GM-2, and GM-3 fabrics for graphene and MXene loading; (**b**) evolution of surface thermal radiation temperatures corresponding to C-1, GM-1, GM-2, and GM-3 fabrics during the heating process (on a hot plate at 100 °C); (**c**) reduction of the radiation temperature of C-1, GM-1, GM-2, and GM-3 fabrics; (**d**) IR images of C-1, GM-1, GM-2, and GM-3 fabrics over a 5 min period; (**e**) IR and optical images of GM-3 fabric covering the palm and back of the hand; (**f**) infrared thermal imaging images made of camouflage clothing, inset is optical image; (**g**) thermal radiation temperature change curves of different color blocks; (**h**) emissivity of different color blocks; and (**i**) schematic of thermal camouflage mechanism of graphene/MXene coating.

The excellent thermal camouflage performance of the graphene/MXene-modified fabrics makes them promising candidates for various practical applications. The graphene/MXene fabrics mostly depend on the loading capacity of MXene and graphene. As the number of loadings increased and the unit weights of MXene and graphene rose, the emissivity of the modified fabrics dropped sequentially (Figure 4a). The progression of equivalent surface thermal radiation temperature for the C-1, GM-1, GM-2, and GM-3 fabrics is displayed in Figure 4b. The fabrics are heated on a heating table at 100 °C (the surface thermal radiation temperature of the heating table is 95 °C). The greatest surface radiation temperature of the C-1 cloth was about 88.8 °C, indicating that it had virtually negligible insulating power against thermal radiation. The surface temperatures of all the fabrics reached their maximum values around 30 s and remained in this range. The surface radiation temperatures of GM-1, GM-2, and GM-3 fabrics are 48.6 °C, 43.3 °C, and 41.9 °C, respectively. Compared to C-1 fabrics, the insulation from thermal radiation is significant, and the surface radiation temperature is reduced, aligning with a decrease in the IR emissivity of the fabrics. Figure 4c shows that the graphene/MXene-modified fabrics have excellent thermal radiation-blocking capabilities. The temperature drop of thermal radiation on the surface of the C-1 fabric is only 6.2 °C, whereas that of the GM-1, GM-2, and GM-3 fabrics reaches 46.4 °C, 51.7 °C, and 53.1 °C, respectively.

There was no change in thermal imaging within 1 min and no temperature change until 5 min later. This shows that the GM-3 fabric has a fast temperature response and satisfactory thermal stability, as demonstrated by Figure 4d (Figure S4 shows an optical image of GM-3 fabric). The IR and optical images of the GM-3 (Figure e1) and C-1 (Figure e2) fabrics covering the palm and back of the hand are displayed in Figure 4e. The GM-3 fabric placed on the palm has a surface radiation temperature of 25.4 °C, which is 10.4 °C lower than the hand’s maximum temperature (35.8 °C) and only 2.4 °C higher than the ambient temperature. The sample placed on the back of the hand has a surface radiation temperature of 25.5 °C, which is 2.5 °C higher than the ambient temperature. The extraordinary flexibility of GM-3 fabric allows it to be cut into a variety of shapes, as shown in Figure S5. These results show that the GM-3 fabrics can effectively achieve a satisfactory IR stealth effect, and the isolation of thermal radiation has the same thermal insulation (Figure S6).

As shown in Figure 4f, the camouflage clothing obtained by scraping green, yellow, and tan paint on the GFM-3 fabric is placed on the heating table at 40 °C, and the infrared thermal imaging of different color blocks shows different colors and is clearly distinguished, which is more in line with the requirements of infrared camouflage. Because the conditions of infrared camouflage are complex, the color displayed by thermal imaging is also different; if the thermal imager of the infrared stealth fabric worn on the body is one color, it can also show human shape, which is not conducive to infrared camouflage. Figure 4g shows the thermal radiation temperature change curve of different color blocks, which is consistent with the emissivity of different color blocks at 8–12 μm (Figure 4h). The temperature reaches its maximum value in about 50 s, which means that wearing the stealth fabric quickly blends into the complex environment around you.

The primary factor contributing to the GM-3 fabric’s excellent thermal camouflage performance is its low IR emissivity (Figure 4i). However, the effect on the actual temperature is minimal and unimportant, especially when the fabric is used for prolonged high-temperature thermal camouflage (Figure S7). Only a small amount of the thermal radiation energy is released upon contact with the graphene/MXene layer, as MXene blocks most of the radiation energy. From ε + ρ + τ = 1 (where ε, ρ, τ represent emissivity, reflectivity, and transmittance, respectively), given that graphene, MXene, and the modified fabrics are opaque black materials, it can be determined that the transmittance (τ) is 0. Consequently, the lower the emissivity of the graphene/MXene-modified fabrics, the higher the reflectivity and the greater the potential for heat radiation emitted from the external environment to be reflected.

### 3.3. Electrothermal Properties of Graphene/MXene-Modified Fabrics

**Figure 5:**
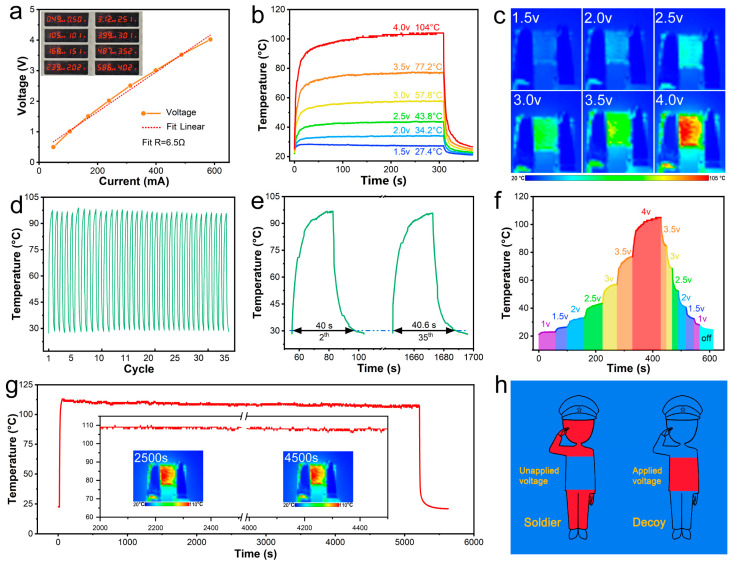
Electrothermal properties of graphene/MXene-modified fabrics. (**a**) V–I linear curve and digital test photos; (**b**) surface temperature profiles of modified fabrics at different voltages; (**c**) thermal imaging of modified fabrics at different voltages; (**d**) stability test curve of modified fabric at 4 V for 35 cycles; (**e**) cycle 2 and 35th cycle curves in the cyclic stability test curves; (**f**) curve of saturation radiation temperature change during voltage adjustment; (**g**) thermostatic stability test curves of modified fabrics connected to a circuit at 4 V (inset shows temperature graphs and thermal images from 2000 s to 2500 s and 4000 s to 4500 s); (**h**) schematic of thermal camouflage of modified fabrics.

In the war, excellent electric heating can not only play a heating role but also allow the dummy to be clearly displayed under the enemy’s infrared thermal imager so as to achieve the effect of confusing the enemy. The electrical–thermal conversion characteristics and durability of graphene/MXene-treated fabrics at varying voltages are displayed in Figure 5. According to the fitted I–V linear curve (Figure 5a), which has a high degree of fit (6.5 Ω), the electrical and thermal characteristics of the modified fabrics essentially follow Ohm’s law and may operate steadily under electrified conditions.

At varying voltages, fabrics heat up quickly to varied temperatures (Figure 5b). At 1.5, 2.0, 2.5, 3.0, 3.5, and 4.0 V, the saturation temperatures were 27.4 °C, 34.2 °C, 43.8 °C, 57.8 °C, 77.2, and 104 °C, respectively. The temperature was comfortable at 2.0 V, and it takes about 9.4 W of energy to bring a warrior uniform to that temperature (Equation S2). In under 30 s, the temperature rose to 91.7 °C at 4 V. Once the voltage was cut, the temperature decreased from 104 °C to 35 °C in 16 s. This suggests that the altered fabric can quickly increase and decrease in temperature, which is crucial for real-world applications, especially in military conflicts. The evidence provided by the IR thermal image (Figure 5c) suggests that the heat distribution is quite uniform, meaning that the slurry is distributed uniformly throughout the scraping and spraying operations.

Long-term charge and discharge stability is achieved by the GM-3 fabric at a voltage of 4.0 V (Figure 5d,e). When powered on for heating, the temperature rises from 30 °C to the highest temperature, and then the power is turned off to further reduce the temperature to 30 °C—this is a loop. The temperature curve of GM-3 fabric in the thirty-fifth cold and hot cycle is almost the same as that in the second cycle, further proving the stability and reliability of GM-3 fabric as a heating device. In addition, no difference is observed in the time it takes to reach the maximum temperature from the start of heating.

In practical applications, the fast responsiveness of the modified fabric to continuous voltage changes and low-voltage drive is safe and convenient. This is evidenced by the considerable variations in the temperature distribution of the GM-3 fabric in the 0–4 V and 4–0 V voltage ranges (Figure 5f). The altered textiles’ thermal stability at 4 V is displayed in Figure 5g. The temperature remained essentially constant, as shown by the temperature profile and thermal photos taken at 2500 and 4500 s. It remains nearly constant and stabilizes at 108 °C, as shown by the temperature curves and thermal pictures taken at 2500 and 4500 s. When the power supply is removed (Figure S8), the temperature quickly drops to about 20 °C.

The graphene/MXene-modified fabric’s thermal camouflage is depicted in Figure 5h. In general, the heat radiation emitted by people’s bodies is significantly higher than the surrounding environment, which is detected in the thermal imaging equipment. This is not conducive to the concealment of the warrior. When the modified fabric is covered in the body, the heat radiation emitted by the human body is reflected back by the modified fabric so that in the thermal imaging camera, the warrior and the surrounding environment become one to achieve the role of stealth. In addition, when we need to confuse the enemy, we can wear the modified fabric on the dummy body, which has electric heating, so that the heat radiation emitted is similar to the human body and higher than the heat radiation emitted by the surrounding environment so that the enemy’s thermal imaging camera can be clearly found in the false target set up by our side, thus playing a role in confusing the enemy. In addition, it can keep soldiers or equipment warm in extremely cold conditions. Therefore, fabrics treated with graphene and MXene show outstanding Joule heating and thermal stability at low voltage, making them practical and workable for outdoor thermal decoying, warming, and camouflage.

### 3.4. Electromagnetic Shielding Properties of Graphene/MXene-Modified Fabrics

**Figure 6:**
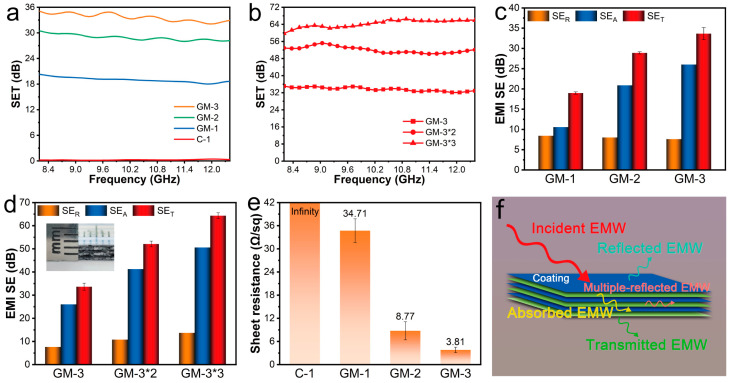
Electromagnetic shielding performance of MXene-modified IR stealth fabrics in the X-band. (**a**) EMI shielding performance of C-1, GM-1, GM-2, and GM-3 fabrics in X-band; (**b**) EMI shielding performance of GM-3 fabrics with different numbers of layers; (**c**) mean values of SE_R_, SE_A,_ and SE_T_ in X-band for GM-1, GM-2, and GM-3 fabrics; (**d**) mean values of SE_R_, SE_A_, and SE_T_ in X-band for GM-3 fabrics with different layers (*n stands for n layers); (**e**) square resistance of C-1, GM-1, GM-2, and GM-3 fabrics; and (**f**) schematic diagram of electromagnetic interference shielding mechanism of graphene/MXene coating.

The GM-1, GM-2, and GM-3 fabrics have varying average shielding efficiencies, corresponding to 19 dB, 28.9 dB, and 33.6 dB, respectively. However, the C-1 cloth lacks any electromagnetic shielding feature (Figure 6a). Due to the numerous internal reflections and absorptions of electromagnetic waves between the fabric layers, the electromagnetic shielding efficiency of two- and three-layer fabrics can be further enhanced through the simple superposition of fabric layers (Figure 6b), with average shielding efficiencies of 52.1 and 64.3 dB, respectively, protecting public health from electromagnetic radiation pollution.

It is well known that for EMI shielding materials, the total EMI SE (SE_T_) is the sum of reflections (SE_R_), absorptions (SE_A_), and multiple reflections (SE_M_), and SE_M_ is usually ignored when SE_total_ > 15 dB [40]. As shown in Figure 6c,d, the fabrics’ absorption value (SE_A_) surpasses their reflection value (SE_R_). This is because when an electromagnetic wave strikes MXene, it undergoes internal reflection, being absorbed and reflected numerous times between MXene sheets, graphene sheets, and MXene and graphene sheets. Significant amounts of electromagnetic wave energy are expended during this process, enhancing the modified fabric’s superior electromagnetic shielding capabilities. Owing to the significant consumption of electromagnetic wave energy during this procedure, the changed fabric performs exceptionally well in electromagnetic shielding. The multilayer MXene-modified fabrics’ surface electromagnetic reflectivity (SE_R_), surface electromagnetic absorption (SE_A_), and surface electromagnetic transmission (SE_T_) values further demonstrate that while the SE_R_ value remains relatively constant, the SE_A_ and SE_T_ exhibit a notable rising trend as the number of layers increases. This is because stacking GM-3 fabrics increases the number of MXene and graphene coatings. These flakes increase the number of times electromagnetic waves are absorbed and reflected internally to dissipate energy. This process further improves the electromagnetic shielding capabilities of the MXene-modified fabrics. This is because as the number of layers of GM-3 fabric increases, more MXene and graphene flakes are produced. This increases the number of electromagnetic wave reflections and absorptions and causes the energy generated by these waves to be absorbed. The inset completely illustrates the lightweight and thinness of the graphene/MXene-modified fabric, displaying the three-layer GM-3 fabric’s thickness of only 2 mm.

An object’s resistance is correlated with how well it shields. The GM-1, GM-2, and GM-3 square resistances decrease in the order of 34.71, 8.77, and 3.81 Ω/sq, respectively, as illustrated in Figure 6e, aligning with the reports [41] that the object’s resistance has an impact on how well it shields against electromagnetic interference. When an incident electromagnetic wave is directed toward the surface of the MXene coating, numerous free charges on the surface cause high electrical conductivity; thus, some of the incident wave is reflected out, while the remaining incident electromagnetic wave penetrates the graphene/MXene coating. Due to a continuous conductive network structure within the coating, which allows penetrating electromagnetic waves to be reflected multiple times, only a small amount of electromagnetic waves pass through the graphene/MXene coating, effectively shielding against electromagnetic interference. This demonstrates the electromagnetic interference shielding mechanism of the GM-3 fabric (Figure 6f).

## 4. Conclusions

The modified fabric exhibits superior IR stealth, electrothermal, and electromagnetic shielding properties by applying graphene paste scratched onto an alkali-treated cotton fabric and spraying MXene on it. This study utilized graphene paste to act as a transverse thermal conductive layer and reduce the surface resistance of the fabrics. MXene was used as an IR low-emissivity layer to reduce the IR emissivity of the modified fabric surfaces. The modified fabrics have very low infrared emissivity in the 3–5 and 8–14 μm bands, and in the 100 °C heating table (with a surface radiation temperature of 95 °C), the surface temperature reduction was more than 53 °C. In addition, the graphene/MXene-modified fabrics have good Joule heat (4V, 91.7 °C) and electromagnetic interference shielding (64.3 dB) properties. It has enormous application potential in the fields of smart wearable materials and the military due to its easy preparation, industrial production, and broad applicability.

## Figures and Tables

**Table 1:** Sample types and preparation method.

Samples	Preparation Method
C-1	Pre-treated cotton twill fabric
G-1	Scrape graphene paste onto C-1 fabric
M-1	Spray MXene aqueous suspension on C-1 fabric
GM-1	The C-1 fabric is scraped with graphene paste and then sprayed with MXene water-based suspension
GM-2	The GM-1 fabric is scraped with graphene paste and then sprayed with MXene water-based suspension
GM-3	The GM-2 fabric is scraped with graphene paste and then sprayed with MXene water-based suspension

## Data Availability

Data will be made available on request.

## Supplementary Materials

The following supporting information can be downloaded at: https://www.mdpi.com/article/10.3390/nano15020098/s1, Figure S1: (a) distribution of tensile strength and elongation length at break of C-1 and GM-3 fabrics; (b) mean and standard deviation of tensile breaking strength of fabric; Figure S2: Fastness test of GM-3 fabric; Figure S3: Ti 2p high-resolution spectra of GM-3 fabric; Figure S4: Optical image of GM-3 fabric placed on a heating table; Figure S5: IR image of GM-3 fabric cropped into QDU shape, inset is optical image; Figure S6: Simple insulation test; Figure S7: Comparison of GM-1, GM-2, GM-3 fabrics temperature; Figure S8: Temperature change curve of GM-3 fabric when voltage is turned off.
