# Supplementary material for: A Graphene/MXene-Modified Flexible Fabric for Infrared Camouflage, Electrothermal, and Electromagnetic Interference Shielding
Source: Nanomaterials (Basel). 2025 Jan 9;15(2):98. doi: 10.3390/nano15020098 (PMC11767746; doi:10.3390/nano15020098)
Supplement: Supplementary file 1 [file nanomaterials-15-00098-s001.zip › nanomaterials-3396240-supplementary.pdf]

Supporting information

A Graphene/MXene-Modified Flexible Fabric for Infrared Camouflage, Electrothermal, and Electromagnetic Interference Shielding

Xianguang Hou[1], Ziyi Zang[1], Yaxin Meng[1], Tian Wang[1], Shuai Gao[1], Qingman Liu[1], Lijun Qu[1] and Xiansheng Zhang[1,*]

[1] Shandong Key Laboratory of Medical and Health Textile Materials, College of Textiles and Clothing, State Key Laboratory of Bio-Fibers and Eco-Textiles, Research Center for Intelligent and Wearable Technology, Qingdao University, Qingdao 266071, P. R. China

* Correspondence：xshzhang@qdu.edu.cn

The Stefan-Boltzmann law states the total power radiated per unit area of the surface of a blackbody (i.e., an ideal surface that absorbs all incident thermal radiation) in a unit of time is proportional to the fourth power of the thermodynamic (absolute) temperature of the blackbody:

$$E = \varepsilon\sigma \mathrm{T}^4 \qquad \text{(Equation S1)}$$

Where E is the radiant exitance or emittance of a black body, measured in watts per square meter (W/m^2); ε is the emissivity of the surface, a dimensionless number between 0 and 1, which indicates how closely the object's emission behavior approaches that of an ideal black body; σ is the Stefan-Boltzmann constant, approximately equal to 5.67×10^{-8} W/(m^2·K^4). T is the absolute temperature of the body in kelvins (K).

The experimental results show that we apply the voltage at both ends of the sample of 1×2 cm size, and the experimental data show that the voltage is 2.02 V and the current is 239 mA, and at this time, the surface temperature is 34.2℃, which is in line with the range of the human body's comfortable temperature. From the power formula:

$$P = V\times I \qquad \text{(Equation S2)}$$

where V is the voltage and I is the current, $P_1 \approx 0.485$ W. It is estimated that about 40 of these nuggets would suffice to heat the critical parts of a warrior's uniform, so the total power is $P_{total} \approx P_1\times40 = 19.4$ W, so, theoretically, the activation of a set of soldier's uniforms requires about 19.4 W of energy.

EMI shielding performance of SE_R, SE_A and SE_T according to the following equation:

$$R = |S_{11}|^2 \qquad \text{(Equation S3)}$$

$$T = |S_{21}|^2 \qquad \text{(Equation S4)}$$

$$SE_R = -\log_{10}(1-R) \qquad \text{(Equation S5)}$$

$$SE_A = -\log_{10}\left(\frac{T}{1-R}\right) \qquad \text{(Equation S6)}$$

$$SE_T = SE_R + SE_A \qquad \text{(Equation S6)}$$

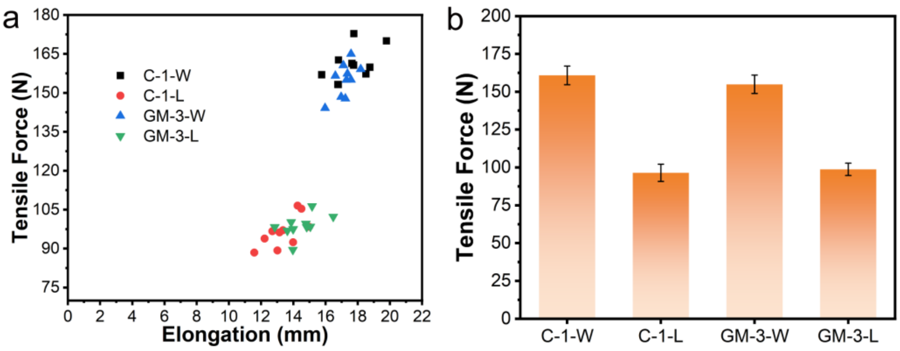

Figure S1. (a) distribution of tensile strength and elongation length at break of C-1 and GM-3 fabrics; (b) mean and standard deviation of tensile breaking strength of fabric.

Figure S1a shows the distribution of breaking tensile strength and elongation length of C-1 and GM-3 fabrics in the same longitude and latitude direction. The distribution of warp and latitude of C-1 fabric is similar to that of GM-3 fabric respectively, which can also be reflected in Figure S1b. This shows that the mechanical properties of C-1 and GM-3 fabrics are the same, and the loading of graphene and MXene has no effect on the mechanical properties of the original fabric.

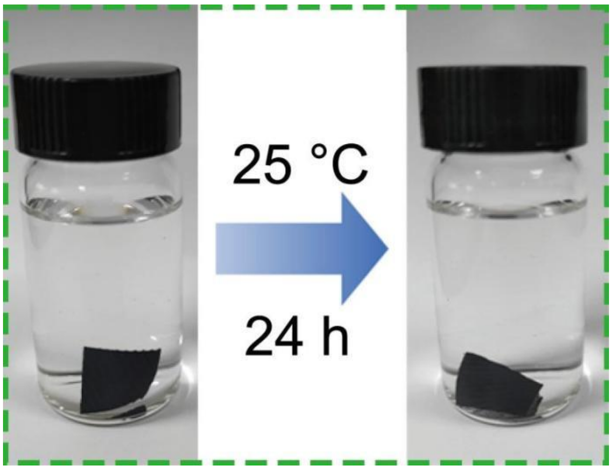

Figure S2. Fastness test of GM-3 fabric.

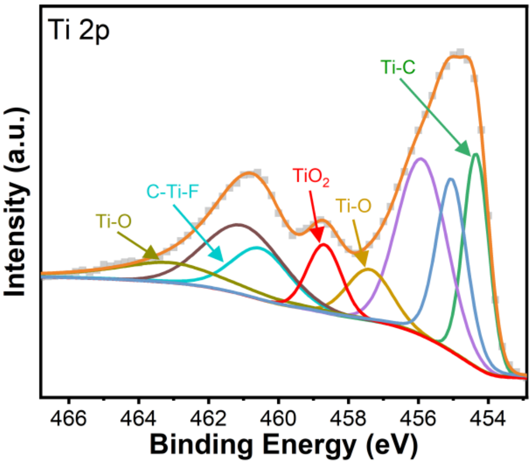

Figure S3. Ti 2p high-resolution spectra of GM-3 fabric.

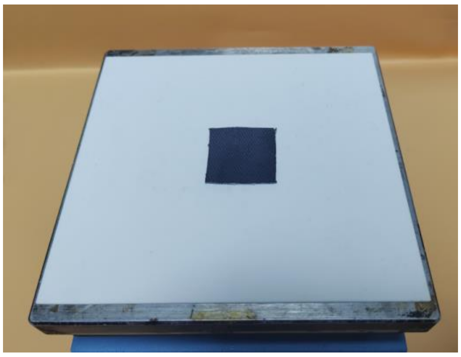

Figure S4. Optical image of GM-3 fabric placed on a heating table.

Fig. S4 shows an optical picture; the surface of the heating table is covered with a layer of ceramic plate, ensuring even heating. As the heat from the process causes the fabric to curl, the two sides of the fabric change over, resulting in a drop in the temperature and an uneven surface temperature of the C-1 cloth.

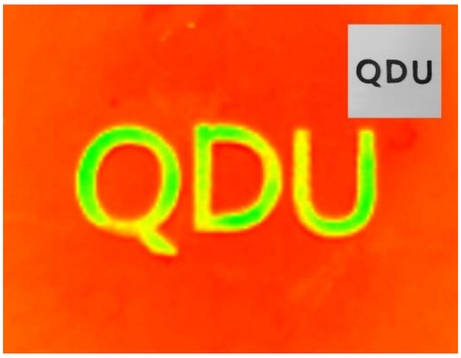

Figure S5. IR image of GM-3 fabric cropped into QDU shape, inset is optical image.

The GM-3 fabric's exceptional flexibility allows it to be cut into various shapes. The potential of IR stealth is demonstrated by the letters "QDU," which are cut from GM-3 fabric and visible when placed on a hot plate with a surface radiant temperature of 95°C, as depicted in Figure 4e (Inset is an optical image).

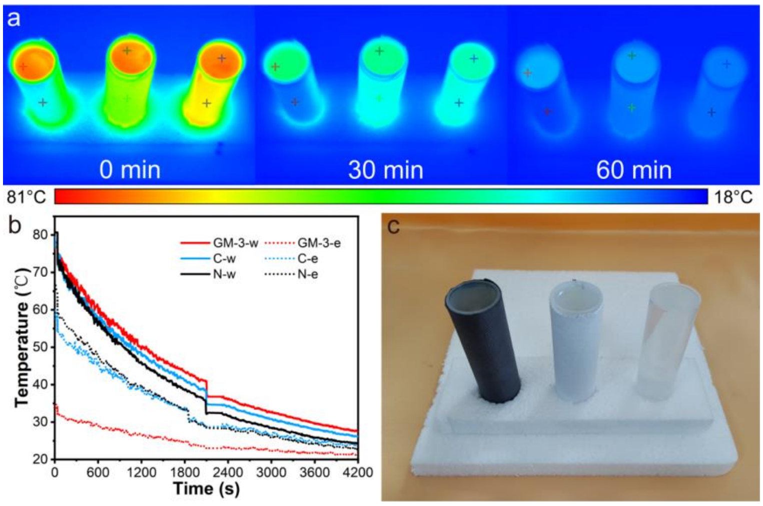

Figure S6. Simple insulation test. a) Thermal imaging images of GM-3 fabric-wrapped centrifuge tube (left), C-1 fabric-wrapped centrifuge tube (middle), and non-fabric-wrapped centrifuge tube (right) at the beginning of hot water pouring, at 30 minutes, and at 60 minutes; b) Thermal radiation temperature curves of the mouth and wall of the three centrifugal tubes; c) Simple heat insulation test device.

The GM-3 fabric and C-1 fabric were respectively wrapped on the outer side of the centrifugal tube wall, and 95°C hot water was poured into the centrifugal tube to observe the change of thermal radiation temperature under the thermal imager. As

shown in Fig. S5a, the radiation temperature of the outer wall of the centrifuge tube wrapped with GM-3 fabric is the lowest, while that of the outer wall of the unwrapped centrifuge tube is the highest, which indicates that GM-3 fabric has a significant inhibitory effect on the heat loss of hot water in the centrifuge tube. This can also be reflected in Fig. S5b. The water temperature at the mouth of the centrifuge tube wrapped with GM-3 fabric decreased the most slowly, but the temperature changed greatly within 70 min because the amount of water was only 50 mL, and the heat was mainly lost by the mouth of the centrifuge tube. Fig. S5c is a simple thermal insulation test device with centrifugal tubes placed on foam to reduce the rate of heat loss from the bottom.

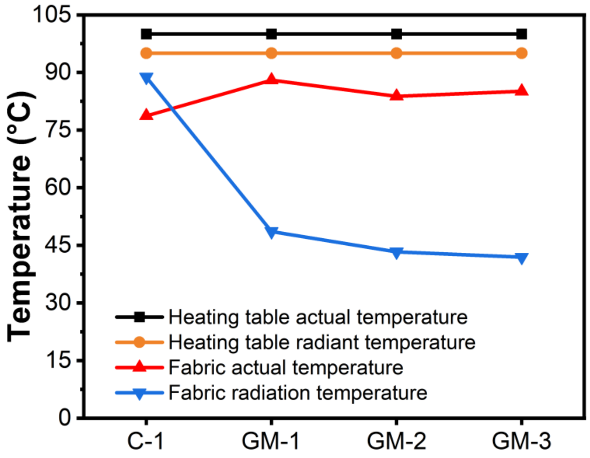

Figure S7. Comparison of GM-1, GM-2, GM-3 fabrics temperature.

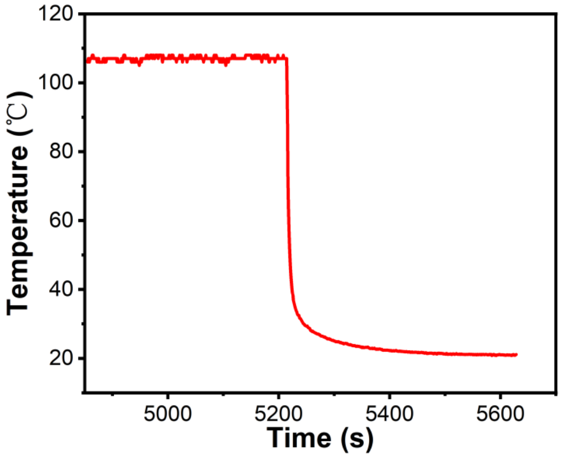

Figure S8. Temperature change curve of GM-3 fabric when voltage is turned off.
